# Stability of smooth and rough mini-implants: clinical and biomechanical evaluation - an *in vivo*study

**DOI:** 10.1590/2177-6709.20.5.035-042.oar

**Published:** 2015

**Authors:** Giselle Naback Lemes Vilani, Antônio Carlos de Oliveira Ruellas, Carlos Nelson Elias, Cláudia Trindade Mattos

**Affiliations:** 1 PhD in Orthodontics, Universidade Federal do Rio de Janeiro (UFRJ), Rio de Janeiro, Rio de Janeiro, Brazil; 2 Professor of Orthodontics, Universidade Federal do Rio de Janeiro (UFRJ), School of Dentistry, Rio de Janeiro, Rio de Janeiro, Brazil; 3 Professor, Instituto Militar de Engenharia, School of Engineering, Department of Material Sciences, Rio de Janeiro, Rio de Janeiro, Brazil.; 4 Professor of Orthodontics, Universidade Federal Fluminense (UFF), School of Dentistry, Niterói, Rio de Janeiro, Brazil.

**Keywords:** Orthodontic anchorage procedures, Osseointegration, Orthodontics

## Abstract

**Objective:**

To compare *in vivo* orthodontic mini-implants (MI) of smooth (machined) and rough (acid etched) surfaces, assessing primary and secondary stability.

**Methods:**

Thirty-six (36) MI were inserted in the mandibles of six (6) dogs. Each animal received six (6) MI. In the right hemiarch, three (3) MI without surface treatment (smooth) were inserted, whereas in the left hemiarch, another three (3) MI with acid etched surfaces (rough) were inserted. The two distal MI in each hemiarch received an immediate load of 1.0 N for 16 weeks, whereas the MI in the mesial extremity was not subject to loading. Stability was measured by insertion and removal torque, initial and final mobility and by inter mini-implant distance.

**Results:**

There was no statistical behavioral difference between smooth and rough MI. High insertion torque and reduced initial mobility were observed in all groups, as well as a reduction in removal torques in comparison with insertion torque. Rough MI presented higher removal torque and lower final mobility in comparison to smooth MI. MI did not remain static, with displacement of rough MI being smaller in comparison with smooth MI, but with no statistical difference.

**Conclusions:**

MI primary stability was greater than stability measured at removal. There was no difference in stability between smooth and rough MI when assessing mobility, displacement and insertion as well as removal torques.

## Abstract (pt)

**Objetivo:**

comparar, *in vivo*, mini-implantes (MI) com superfície lisa (usinada) e porosa (tratada com ácido), avaliando sua estabilidade primária e secundária.

**Métodos:**

trinta e seis MI foram inseridos na mandíbula de seis cães, e cada animal recebeu seis MI. Na hemiarcada direita, foram inseridos três MI sem tratamento da superfície (liso); na esquerda, outros três com a superfície tratada com ácido (poroso). Os dois MI distais de cada hemiarcada receberam carga imediata de 1,0N durante dezesseis semanas, e o MI da extremidade mesial não recebeu carregamento. A estabilidade foi medida pelos torques de inserção e de remoção, pela mobilidade inicial e final, e pela distância inter-MI.

**Resultados:**

não houve diferença estatística do comportamento entre os MI lisos e porosos. No entanto, observou-se torque de inserção elevado e mobilidade inicial reduzida em todos os grupos. Para todos os grupos, houve redução dos torques de remoção, em relação ao de inserção. Os MI porosos apresentaram maior torque de remoção e menor mobilidade final, em relação aos MI lisos. Os MI não permaneceram estáticos, sendo o deslocamento dos MI porosos menor em relação aos MI lisos, mas sem diferença estatística.

**Conclusões:**

a estabilidade primária dos MI foi maior do que a estabilidade medida na sua remoção; não houve diferença na estabilidade entre os MI lisos e porosos ao avaliar-se a mobilidade, o deslocamento e os torques de inserção e remoção.

## INTRODUCTION

Various skeletal anchorage systems have been proposed over the last few years with a view to assisting complex treatment and reducing orthodontic treatment time. Mini-implants have produced better results in comparison to other anchorage systems due to being inserted and removed with ease, and particularly due to the reduced size of the devices, which broadens their scope of use.^1^

With a reduction in mini-implant size, the screws are now made of titanium alloy (Ti6Al4V), which increases fracture strength.^2^ The disadvantage of Ti6Al4V alloy is its lower degree of osseointegration and greater susceptibility to corrosion *in vivo*, both of which may hinder stability.^3^

Osseointegration stands for direct contact between bone and implant without interposition of soft tissue layers. It is beneficial since it increases stability and raises success rates of MI as temporary anchorage devices, thus expanding their biomechanical possibilities.^4^ Various factors must be taken into account in order to achieve implant osseointegration, namely: material biocompatibility, implant surface conditions, patient's conditions, the surgical technique employed and the load applied on implants after placement.^5^Studies have shown that surface treatment applied to the active parts of mini-implants result in roughness that favors bone-implant contact.^6^^-^ 9 Acid etching is a simple method that requires little infrastructure and results in implant roughness, making implant surface homogeneous and with a large active surface area that enables better bioadhesion.^10^

At present, there is an increasing trend towards applying immediate loading for orthodontic purposes, particularly because studies have shown that mini-implants are able to bear continuous forces immediately after placement^11^ without hindering anchorage and success rates.^12^ Nevertheless, it is necessary to assess the effects of acid etching on stability of mini-implants subject to immediate loading.

The percentage of bone/mini-implants contact must be adequate in order to bear orthodontic forces and raise stability success rates of temporary anchorage devices; however, it must not be excessive, so as to allow anchorage devices to be removed at the end of treatment without leading to anchorage device or bone fracture.^13^

The aim of this study was to compare *in vivo* orthodontic mini-implants made of Ti6Al4V alloy, with smooth (machined) and rough (acid etched) surfaces, assessing primary and secondary stability.

## MATERIAL AND METHODS

This animal study protocol was approved by the Ethics Committee on Animal Use (CEUA) of the Health Science Center (CCS) of Universidade Federal do Rio de Janeiro, Brazil (protocol: ODONTO 010).

**Figure 1:**
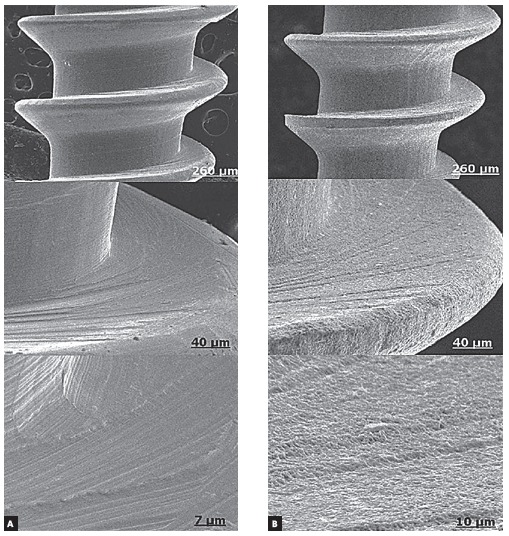
- Electromicrographs of smooth (A) and rough (B) mini-implant surfaces.

**Figure 2:**
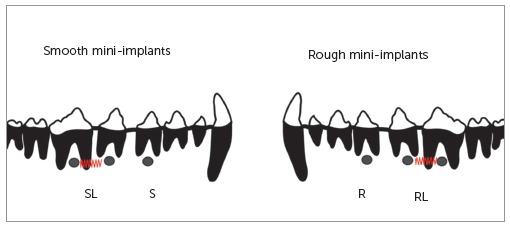
- Diagram showing the position of smooth and rough mini-implants inserted in the external buccal cortical bone and loaded with NiTi springs. SL = Smooth with immediate loading; S = Smooth without load; RL = Rough with immediate loading; R = Rough without load.

Thirty-six (36) mini-implants made of Ti6Al4V alloy (Conexão Sistemas e Próteses, Arujá, SP, Brazil), measuring 1.5 x 6.0 x 2.0 mm, were used in the present research. Of them, 18 had no surface treatment (smooth) while 18 were subject to acid etching specifically carried out for this study. To this end, an aqueous solution made of nitric acid (HNO_3_), hydrochloric acid (HCl) and sulfuric acid (H_2_SO_4_) (rough standard by Conexão) (Fig 1) was used. Six (6) adult male mongrel dogs weighing approximately 18.0 kg were used. Each animal had six (6) mini-implants placed buccally between roots in the alveolar bone of the mandible. On the right side, three smooth mini-implants were inserted, whereas on the left side, three rough mini-implants were inserted. The two distal mini-implants were subject to immediate load while the mesial extremity remained without loading. Mini-implants were divided into four groups: S = smooth without load; SL = smooth with immediate load; R = rough without load; RL = rough with immediate load. Figure 2 discloses a diagram illustrating the position of smooth and rough mini-implants.

**Figure 3:**
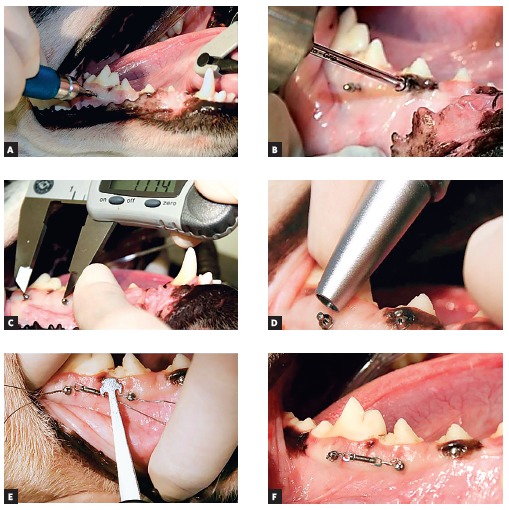
- Photographs illustrating the steps for mini-implant placement: A) Initial mini-implant placement with manual key; B) Conclusion of placement with torque wrench; C) Measurement of inter mini-implant distance; D) Use of Periotest; E) Measurement of force of 1 N; F) NiTi spring in position.

After initial dental prophylaxis, radiographs were taken by means of the parallelism technique and with the aid of an acrylic positioner, so as to check for space availability between roots. Subsequently, the gingiva was marked by a millimetric periodontal probe located as closely as possible to the limit between keratinized and non-keratinized gingiva in the region of root bifurcation of third and fourth premolars and first molar. The opening made in the cortical bone for subsequent mini-implant placement was done with the aid of a pilot bur 1.0 mm in diameter (Conexão Sistemas e Próteses, Arujá/SP, Brazil), at a speed of 600 rpm, without pressure and under copious irrigation with 0.9% saline solution. Mini-implants were inserted perpendicular to the buccal cortical surface of the alveolar bone with the aid of a manual key provided by the manufacturer and under clockwise movement concluded just before the two final turns were performed (Fig 3A). Mini-implant insertion was concluded with the manual key coupled to a portable digital torque meter (Instrutherm TQ 680, Korea) used to obtain the maximum insertion torque value (N.cm) (Fig 3B).

The distance between loaded mini-implants was recorded in each quadrant soon after mini-implants were inserted, before fixation of the spring and after a period of 16 weeks. The center of the upper portion of the device head was used as reference. Measurements were performed with a digital caliper (Starret Indústria e Comércio Ltda, São Paulo, Brazil) (Fig 3C).

Mini-implant mobility was clinically assessed at two time intervals: at mini-implant placement and after 16 weeks. Quantitative mobility assessment was performed by Periotest (Medizintechnik Gulden e.K., Modautal, Germany), and consisted of a vibration analysis performed to detect lateral movement of an implant inside the bone. After the instrument was calibrated, it was placed perpendicular to the head of the mini-implant, horizontal towards the ground, with the head of the handpiece placed 2.0 to 3.0 mm from the mini-implant head. Measurements oscillated at a frequency of around four times per second. Results were digitally and audibly shown by a descriptive numerical value and ranged from -8 to +50^14 ^(Fig 3D). Mobility and distance between mini-implants were recorded twice and the mean values obtained.

The two distal mini-implants received loading immediately after insertion. A load of 1.0 N was applied by NiTi springs for 16 weeks. Mesial mini-implants were not subject to loading. The force released by the spring was quantified by a tensiometer (Zeusan, Germany) (Fig 3E). At last, the spring was tied to two mini-implants by a 0.012-in ligature wire (Fig 3F). After the surgical procedures, all animals were subject to anti-inflammatory and analgesic therapy for three days with injectable flunixin meglumine (Schering Plough Indústria Química e Farmacêutica S.A., Rio de Janeiro/RJ, Brazil). The animals were fed with animal food, ground and moisturized with water, suitable for puppies, and were provided with water *ad libitum*. They also received dental prophylaxis performed with a brush and anti-tartar tooth paste (C.E.T.^(r)^ Pasta Enzimática, Virbac, São Paulo, Brazil) once a week during the experiment. Subsequently, the mini-implants were cleaned with 0.12% chlorhexidine gluconate (PerioGard^(r)^, Colgate-Palmolive Indústria Comércio Ltda, São Bernardo do Campo, SP, Brazil). To this end, the dogs were sedated with an intramuscular injection of 0.4 mg/kg xylazine (Bayer S/A, São Paulo, SP, Brazil) and 0.5 mg/kg morphine (União Química Farmacêutica Nacional S/A, São Paulo, SP, Brazil).

By the end of the 16-week period, mini-implants were removed and maximum removal torque was recorded.

### Statistical analysis

Insertion and removal torque, initial and final mobility, and difference in inter mini-implants distance were expressed in means and standard deviation values. Kolmogorov-Smirnov non-parametric test was used to test for normality of the sample. Groups S, SL, R and RL were compared in terms of insertion and removal torque as well as initial and final mobility by means of one-way Analysis of Variance (ANOVA) associated with Tukey post-test to find whether there was significant difference between groups. Student's t-test was used to assess potential differences in inter mini-implants distance.

## RESULTS

Of 36 mini-implants, six were lost during the experiment (one S, three SL, one R and one RL). The success rate of all mini-implants was 83.3%. Rough mini-implants presented a higher success rate (88.8%) when compared to smooth ones (77.7%). The results of the tests performed with two mini-implants were not used due to loss of mini-implants attached to the spring. Assessment was carried out in 28 mini-implants, five from group S, eight from SL, five from R and ten from RL.

When mini-implants performance was compared, no statistically significant difference was found (*p* > 0.05) between groups for any variables (Table 1). High insertion torque and reduced initial mobility values were observed. Conversely, at the end of the experiment, removal torque was low and final mobility was high; with different values were found between smooth and rough mini-implants. Rough mini-implants presented higher secondary stability, with higher removal torque and lower final mobility when compared to smooth mini-implants, but without statistical significance.

Smooth mini-implants presented with higher mean displacement (0.94 ± 1.33 mm) when compared to rough mini-implants (0.39 ± 0.19 mm) at the end of the experiment; however, this difference was not statistically significant (*p* = 0.387) (Table 2).

## DISCUSSION

In the present study, primary stability was assessed quantitatively by insertion torque (IT) and initial mobility (IMb). Mean IT values were high for groups S (19.20 N.cm), SL (18.00 N.cm), R (19.00 N.cm) and RL (15.90 N.cm), with no statistical difference between them. High IT values may be related to greater thickness of dogs' cortical bone,^14^^,^ 15 ^,^ 16 small bone perforation in relation to mini-implants diameter^17^ and deeper mini-implants insertion, with potential compression of the cortical bone by the transmucosal profile.^18^^,^ 19 ^,^ 20 Previous research conducted with dogs have shown similar high IT values in mini-implants subject to surface treatment (15.27 ± 6.65 N.cm) and in smooth mini-implants (19.25 ± 8.34 N.cm) when slightly larger mini-implants (1.8 x 8.5 mm) were used.^8^ Other studies conducted with dogs presented even higher IT values,^19^^,^ 21 with high success rates, which suggests that IT is higher in mini-implants placed in dog's mandibles, which does not necessarily lead to failure.

**Table 1:** - Values of insertion torque, initial mobility, removal torque and final mobility.

		**S**	**SL**	**R**	**RL**
Insertion torque	Mean (SD)	19.20 (1.64)	18.00 (1.19)	19.00 (3.31)	15.90 (2.68)
	Statistics	A	A	A	A
Initial mobility	Mean (SD)	0.40 (1.51)	-0.06 (2.67)	0.30 (1.09)	-0.20 (2.74)
	Statistics	B	B	B	B
Removal torque	Mean (SD)	2.60 (0.89)	2.75(0.70)	4.00 (1.00)	4.10 (1.52)
	Statistics	C	C	C	C
Final mobility	Mean (SD)	13.60 (6.94)	14.56(4.71)	8.70 (10.42)	7.90 (8.02)
	Statistics	D	D	D	D

**Table 2:** - Inter mini-implant distance values for smooth and rough mini-implants with load.

	**Difference in mean inter MI distance (SD)**	**Statistical significance (*p*-value) Smooth MI x Rough MI**
SL	0.94 (1.33)	0.387
RL	0.39 (0.19)	

In the present study, mini-implants stability was also quantified by means of Periotest used to detect mobility.^22^ The index measured by Periotest varies on a scale ranging from -8 to +50, with values between -8 and +9 indicating that teeth are fixed or implants osseointegrated; between +10 and +19, palpable mobility; between +20 and +29, visible mobility; and between +30 and +50, mobility caused by pressure of the tongue or lip.^14^ In the present study, all groups presented with adequate primary stability, with reduced IMb values (-0.06 to 0.40) in all devices, which suggests absence of mobility. Similar results were obtained by Cha et al^22^ when using mini-implants in dogs. Studies have demonstrated a negative correlation between IT and IMb;^14^^,^ 22 in other words, high IT values and reduced IMb indicated adequate primary stability.

Secondary stability was assessed by removal torque (RT), final mobility (FMb) and difference in inter mini-implants distance. High RT and reduced FMb indicate adequate secondary stability. In the present study, RT was much lower than IT, a behavior that may be associated with peri-implant inflammation caused by biofilm accumulation.^23^ Data available in the literature reveal that reduced IT values are more favorable to achieve osseointegration than high values. In addition, the latter may lead to a high level of compression, which causes local ischemia and bone necrosis at the bone/mini-implants interface, thereby leading to reduction in osseointegration.^24^ However, a recent systematic review found no evidence that a specific IT value is associated with high success rates of orthodontic mini-implants.^25^ Although there were no statistically significant differences between groups, RT values in the rough groups (R and RL) were higher than those of the smooth groups (S and SL), thereby suggesting that acid etching may increase osseointegration success rates. Klokkevold et al^26^ found RT values to be four times higher in mini-implants subject to acid etching when compared to machined surfaces, after waiting eight weeks for load application.

Several authors have considered it essential to wait for healing in order to increase the potential for osseointegration.^8^^,^ 9 ^,^ 27 However, when comparing the resistance of mini-implants subject to surface treatment in five different periods of loading, Mo et al^23^ found high RT values in mini-implants immediately loaded and similar success rates in all periods, which suggests that mini-implants may be immediately loaded. Therefore, in the present study, immediate loading was used, since it is a trend in Orthodontics, bearing in mind that various researches^11^^,^ 12 ^,^ 14 ^,^ 23 ^,^ 31 have proved it to be effective.

In the present study, FMb values were higher than IMb ones. These results are in agreement with data found in the literature, showing that secondary stability of smooth mini-implants is lower in comparison to primary stability. Rough mini-implants presented better stability at the time of removal; however, without statistical difference. Lower FMb values for rough mini-implants suggest absence of mobility, whereas the higher values for smooth mini-implants suggest palpable mobility. In spite of presenting high FMb values, mini-implants proved stable when subject to continuous orthodontic load throughout the entire experimental period. Studies conducted with dogs' mandibles found lower FMb values in smooth mini-implants, which may be due to shorter experimental periods (12 weeks),^28^ since the devices were exposed to biofilm for a shorter period of time.

In the present research, mini-implants did not remain static, with smooth mini-implants showing higher mean displacement (0.94 ± 1.33 mm) than rough mini-implants (0.39 ± 0.19 mm) after load application for sixteen (16) weeks, without statistical differences. Oynarte et al^7^ also found more significant displacement of smooth mini-implants (0.51 mm) in comparison to rough ones (0.12 mm). Similar displacement (0.44 mm) was found after a two-week period,^29 ^in addition to absence of displacement in mini-implants subject to surface treatment after a six-week waiting period.^29^ Studies applying immediate load found a variation that ranged from 0.53 mm^12^ to 0.78 mm^30^ for smooth mini-implants. High displacement values were found in a study applying immediate load (2.2 mm),^31^ but the authors applied elevated forces (6 N) to short mini-implants (3.0 mm). The similarity of displacement values found in the present study and in other studies that used immediate loading to those found in researches that waited for healing before load application indicates that immediate loading can be safely used.

In the present study, mini-implants were removed at the end of the experiment by means of movements applied in anti-clockwise direction. The same results were achieved by Kim et al^27^ and Favero et al^13^ when removing osseointegrated mini-implants larger in diameter.

## CONCLUSIONS

1. The success rate of rough mini-implants (88.8%) was higher than that of smooth mini-implants (77.7%).
2. Primary stability achieved by the end of Ti6Al4V mini-implants placement was higher than stability observed sixteen (16) weeks after insertion.
3. There was no difference in stability between smooth and rough mini-implants when assessing mobility, displacement and the insertion as well as removal torque.
